# Cost-consequence of abatacept as first-line therapy in Japanese rheumatoid arthritis patients using IORRA real-world data

**DOI:** 10.1371/journal.pone.0277566

**Published:** 2022-11-16

**Authors:** Eiichi Tanaka, Eisuke Inoue, Ayako Shoji, Jonas Nilsson, Christos Papagiannopoulos, Devender Dhanda, Yuri Yoshizawa, Mai Abe, Kumiko Saka, Eri Sugano, Naohiro Sugitani, Moeko Ochiai, Rei Yamaguchi, Katsunori Ikari, Hisashi Yamanaka, Masayoshi Harigai

**Affiliations:** 1 Division of Rheumatology, Department of Internal Medicine, Tokyo Women’s Medical University School of Medicine, Tokyo, Japan; 2 Department of Rheumatology, Institute of Rheumatology, Tokyo Women’s Medical University Hospital, Tokyo, Japan; 3 Research Administration Center, Showa University, Tokyo, Japan; 4 Medilead Inc., Tokyo, Japan; 5 ICON plc., Stockholm, Sweden; 6 Bristol-Myers Squibb, Lawrenceville, New Jersey, United States of America; 7 Bristol-Myers Squibb, Tokyo, Japan; 8 Department of Orthopedics, Institute of Rheumatology, Tokyo Women’s Medical University Hospital, Tokyo, Japan; 9 Rheumatology, Sanno Medical Center, Tokyo, Japan; 10 Department of Rheumatology, International University of Health and Welfare, Tokyo, Japan; St Marianna University School of Medicine, JAPAN

## Abstract

**Objectives:**

To investigate the cost-effectiveness of abatacept (ABA) as first-line (1L) therapy in Japanese rheumatoid arthritis (RA) patients using data from the Institute of Rheumatology, Rheumatoid Arthritis database.

**Methods:**

A decision-analytic model was used to estimate the cost per American College of Rheumatology response of at least 50% improvement (ACR50) responder and per patient in Clinical Disease Activity Index (CDAI) and Simplified Disease Activity Index (SDAI) remission from a Japanese healthcare payers’ perspective over a 2-year time horizon. Clinical characteristics of patients on ABA-1L were matched with those of patients on ABA second or later line (2L+) or tumour necrosis factor inhibitor (TNFi)-1L directly or using propensity scores. Resource utilisation and medical costs were calculated from the Japan Medical Data Center claims database. Parameter uncertainty was addressed by sensitivity and subgroup analyses (age, treatment duration, Japanese version of Health Assessment Questionnaire [J-HAQ] score).

**Results:**

Incremental costs per member per month (ΔPMPM) for ABA-1L versus TNFi-1L and ABA-2L+ were -1,571 Japanese Yen (JPY) and 81 JPY, respectively. For ABA-1L versus TNFi-1L, ΔPMPM by ACR50 response was -11,715 JPY and by CDAI and SDAI remission 11,602 JPY and 47,003 JPY, respectively. Corresponding costs for ABA-1L were lower for all outcome parameters versus those for ABA-2L+. Scenario analyses showed that ABA-1L was cost-effective over TNFi-1L in patients <65 years for any outcome. Furthermore, ABA-1L was cost-effective over ABA-2L+ for all outcomes in patients with age <65 years, disease duration <5 years and J-HAQ ≥1.5.

**Conclusions:**

ABA-1L demonstrated a favourable cost-effectiveness profile in RA patients, accruing savings for the Japanese healthcare payers.

## Introduction

Rheumatoid arthritis (RA) is a chronic, systemic inflammatory disorder characterised by inflammation and swelling of synovial joints that often progresses to destructive joint disease, joint damage, impaired joint function, pain, tenderness, increasing disability and reduced quality of life. RA yields a substantial economic burden on both direct medical and non-medical costs as well as indirect costs [1]. The treatment of the disease has significantly progressed, especially owing to the launch and increased use of biological disease-modifying antirheumatic drugs (bDMARDs), and remission has become a realistic treatment goal for many patients [2]. Treatment guidelines have recommended abatacept (ABA), a selective T-cell co-stimulation modulator, as an option for use as a first-line (1L) bDMARD in patients with an inadequate response to the conventional DMARD therapy [3, 4]. At the same time, as treatment with bDMARDs is expensive and yields a considerable economic burden for patients and therefore increases the demand of finding cost-effective treatment options, studies focusing on its pharmacoeconomics have been conducted in several countries [5, 6] as well as in Japan [1, 7, 8].

In the phase III AMPLE trial, a non-inferiority trial, ABA was compared with adalimumab in bDMARD-naïve RA patients with background methotrexate (MTX). The efficacy of subcutaneous (SC) ABA versus SC adalimumab was compared in adults with RA for ≤5 years, diagnosed with moderate to high disease activity (disease activity score for 28-joint counts [DAS28] C-reactive protein ≥3.2) at screening, despite treatment with MTX with at least 15 mg/week [9]. ABA tends to be used in high-risk patient populations, such as older patients or those with comorbidities, as it has shown a favourable safety profile among DMARDs [10–13]. In order to induce remission early and maintain it for a long time, cost-effective bDMARD treatment is required in the 1L setting since bDMARDs are associated with high costs [5–8].

Using the data between 2007 and 2011, we previously clarified that bDMARDs, including tocilizumab, were the most cost-effective over patients’ lifetime (in long-term) compared with bDMARDs using only tumour necrosis factor inhibitors (TNFi) [8]. However, the medical environment surrounding RA, such as drug options, drug costs, and tendency of patient’s disease activity, has changed from that at the time of our previous publication [14]. Moreover, the previous study had evaluated lifetime (long-term) cost-effectiveness [8]; however, patients are more interested in short-term cost-effectiveness. Thus, studies focusing on the short-term cost-effectiveness of RA treatments are warranted, although such studies are insufficient. Therefore, we designed this study to compare the short-term cost-effectiveness of abatacept. The pharmacoeconomic properties of the early use of ABA have not been sufficiently evaluated in Japan, and the objective of this study was to assess the cost-effectiveness of ABA-1L in Japanese RA patients using data from the Institute of Rheumatology, Rheumatoid Arthritis (IORRA) database. Two different scenarios in the treatment of RA were analysed:

1. ABA-1L versus TNFi-1L, both with and without MTX
2. ABA-1L versus ABA second or later line (2L+) (ABA-2L+), both with and without MTX

## Methods

### Model and database used

**Fig 1:**
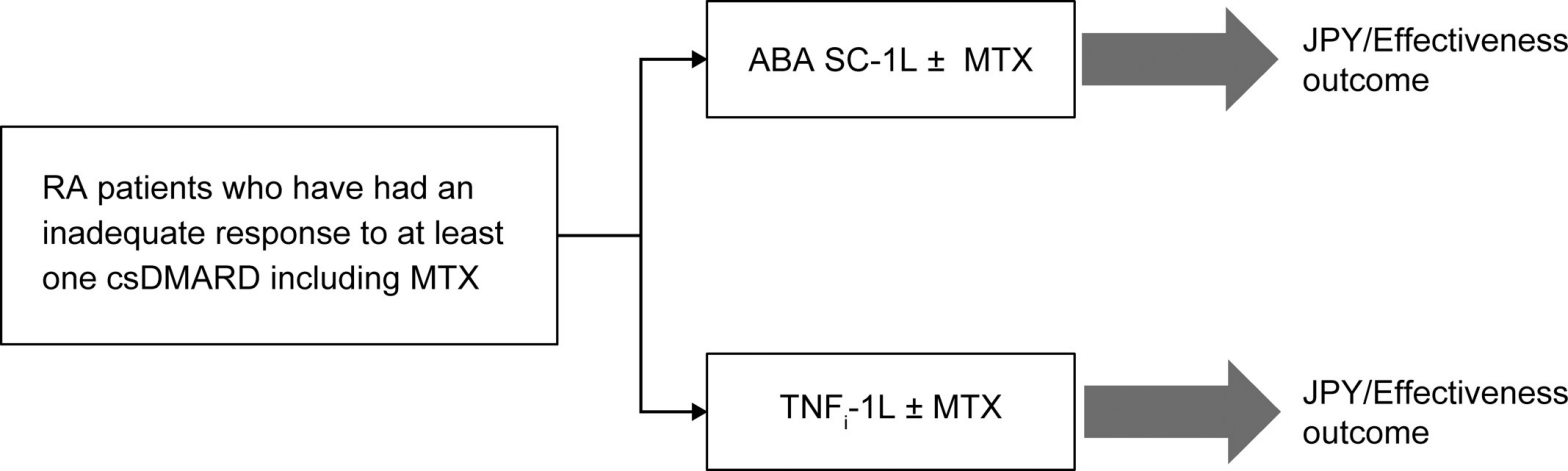
Decision tree structure (ABA-1L vs. TNFi-1L). The target population was RA patients who had an inadequate response to prior treatment with at least one csDMARD, including MTX, in clinical practice. The cost-effectiveness was compared between ABA SC-1L ± MTX and TNFi-1L ± MTX. 1L, first line; ABA, abatacept; csDMARD, conventional synthetic disease-modifying antirheumatic drug; JPY, Japanese Yen; MTX, methotrexate; RA, rheumatoid arthritis; SC, subcutaneous; TNFi, tumour necrosis factor inhibitor.

**Fig 2:**
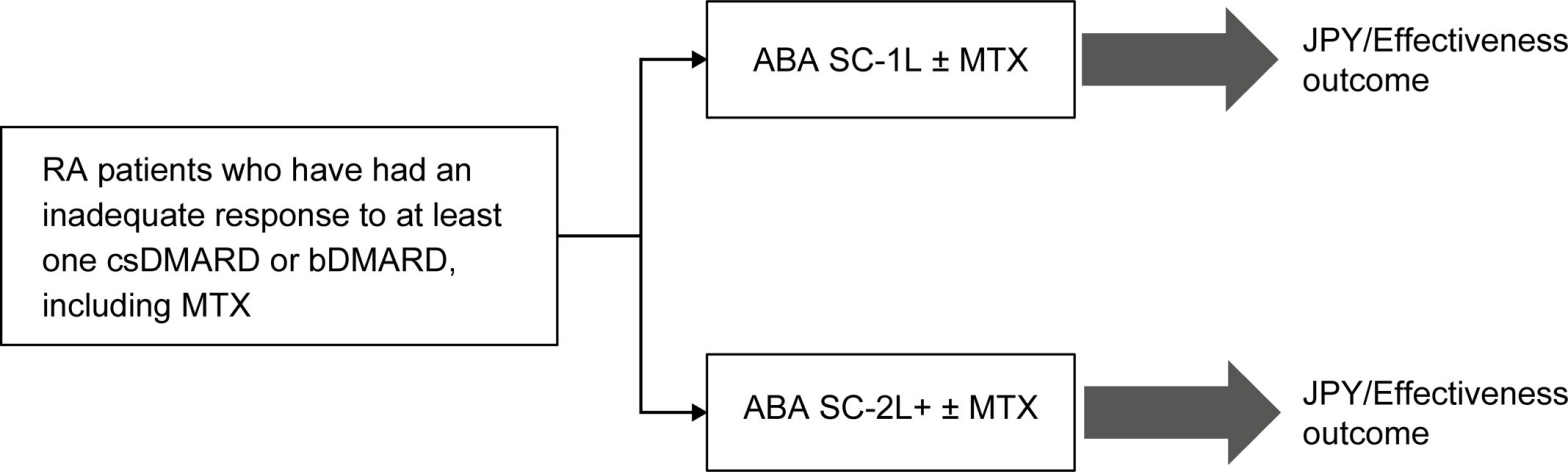
Decision tree structure (ABA-1L vs. ABA-2L+). The target population was RA patients who had an inadequate response to prior treatment with at least one csDMARD or bDMARD, including MTX, in clinical practice. The cost-effectiveness was compared between ABA SC 1L ± MTX and ABA SC 2L+ ± MTX. 1L, first line; 2L+, second or later line; ABA, abatacept; bDMARD, biological DMARD; csDMARD, conventional synthetic disease-modifying antirheumatic drug; JPY, Japanese Yen; MTX, methotrexate; RA, rheumatoid arthritis; SC, subcutaneous; TNFi, tumour necrosis factor inhibitor.

A previously published cost-consequence model that was developed based on the AMPLE study [5] was adapted to the Japanese clinical setting. In the model, the early use of ABA-1L was compared with that of TNFi-1L biologics (Fig 1) and ABA-2L+ (Fig 2), both with and without MTX. In order to adapt the model to the treatment of RA in Japan, de-identified Japanese-specific clinical input data were obtained from the IORRA database (between 1 April 2010 and 30 September 2018). The IORRA database is a single-centre, large-scale, longitudinal observational cohort study of RA patients at the Institute of Rheumatology, Tokyo Women’s Medical University since 2000 [14]. Patients diagnosed with RA who visited our institute were registered in the IORRA cohort, and data were collected biannually. Written informed consent from the patients was obtained at study entry and before each survey. The IORRA cohort study was approved by the ethics committee of Tokyo Women’s Medical University (No. 2922-R16) [14]. Specific data on resource utilisation and costs were collected from commercially available administrative claims data (International Classification of Diseases, Tenth Revision: M05 and M06) from the Japan Medical Data Center Inc (JMDC) database (between 2010 and 2019) [15], which contained approximately 560 million patients’ anonymized data as of June 2018. The database consists of medical check-up and receipts data provided by multiple health insurance societies, which would support the tracking of hospital transfers and/or medications prescribed by different hospitals.

### Matching RA patients in the methods

For the comparison of ABA-1L and TNFi-1L, 95 and 1,501 patients were identified in the IORRA cohort, respectively. Clinical characteristics of patients were matched using propensity scores in order to create a synthetic control arm. We generated propensity scores to predict the probability of a patient initiating ABA by a multiple logistic regression model using the following variables at baseline from the IORRA database: sex, age, J-HAQ, DAS28, disease duration (years), MTX dose (mg), MTX use and comorbidities (myocardial infarction, stroke, hypertension, cardiac failure, pneumonia, interstitial pneumonia, emphysema, peptic ulcer, viral hepatitis, fractures, cancer, depression, diabetes mellitus), where each enrolled patient initiating ABA was matched to a patient initiating TNFi. The baseline characteristics of the identified 82 matched pairs showed no significant difference (Table 1). Standardised differences were less than 0.2 except for the comorbidity of diabetes mellitus (0.305) and C-statistics was 0.79. Accordingly, patients were identified in the JMDC claims database to obtain resource utilisation data; patients whose sex, age and disease duration were matched to the IORRA matched cohort were selected (the observable period is shorter in the JMDC database than in the IORRA cohort, so the disease duration in the former was shorter than that in the latter) (S1 File; S1 Table).

**Table 1:** ABA-1L vs. TNFi-1L: Patient characteristics of matched populations.

	ABA-1L		TNFi-1L		
	Mean, n	SD, %	Mean, n	SD, %	p value^b^
N	82		82		
Gender (female)	76	92.7%	72	87.8%	0.43
Age (yrs)	63.48	12.23	61.17	13.84	0.26
J-HAQ	1.06	0.74	0.97	0.73	0.41
DAS28	4.00	1.11	3.96	1.13	0.80
Disease duration (yrs)	13.51	10.41	14.87	13.12	0.47
MTX dose (mg/week)	6.37	5.57	6.13	5.00	0.67
MTX users	57.00	69.5%	54.00	65.9%	0.74
Comorbidities^a^	44.00	53.7%	43.00	52.3%	-

1L, first line; ABA, abatacept; DAS28, disease activity score for 28-joint counts; J-HAQ, Japanese version of Health Assessment Questionnaire; mg, milligram; MTX, methotrexate; SD, standard deviation; TNFi, tumour necrosis factor inhibitor.

^a^Myocardial infarction, stroke, hypertension, cardiac failure, pneumonia, interstitial pneumonia, emphysema, peptic ulcer, viral hepatitis, fractures, cancer, depression, diabetes mellitus.

^b^McNemar test or paired-samples t-test was used.

For the comparison of ABA-1L versus ABA-2L+, 104 and 159 patients were identified in the IORRA cohort, respectively. To ensure comparability, the following clinical parameters and ranges were used for matching of patients: sex, age (±5 years), DAS28 (±1.0) and disease duration (±5 years). The resulting cohort included 71 matched pairs with no significant difference in baseline characteristics (Table 2), and resource utilisation data were selected from the JMDC database with the same method described above (S2 Table). Lastly, the effectiveness-related outcomes were based on Japanese-specific real-world data from the IORRA database and are presented for ABA-1L versus TNFi-1L (S3 Table) and for ABA-1L versus ABA-2L+ (S4 Table); data used for the scenario analyses (age, disease duration and J-HAQ at index date) are also presented.

**Table 2:** ABA-1L vs. ABA-2L+: Patient characteristics of matched populations.

	ABA-1L		ABA-2L+		
	Mean or n	SD or %	Mean or n	SD or %	p value^a^
N	71		71		
Sex (female)	67	94.4%	67	94.4%	1.00
Age (yrs)	61.6	13	61.0	13.6	0.08
J-HAQ	0.92	0.70	0.95	0.78	0.89
DAS28	3.7	1.0	3.7	1	0.84
Disease duration (yrs)	12.1	8.5	12.6	8.3	0.06
MTX dose (mg/week)	6.6	5.5	7.2	4.5	0.58
MTX users	54	76.1%	58	81.7%	0.42

1L, first line; 2L+, second or later line; ABA, abatacept; DAS28, disease activity score for 28-joint counts; J-HAQ, Japanese version of Health Assessment Questionnaire; mg, milligram; MTX, methotrexate; SD, standard deviation; TNFi, tumour necrosis factor inhibitor.

^a^Fisher’s exact test or student’s t-test was used.

### Model assumption

A time horizon of 2 years was applied in line with the AMPLE trial, and the analysis only considered direct medical costs from the Japanese National Health Service perspective in a hypothetical cohort of 1,000 patients. However, results were presented per member per month (PMPM). Costs based on standard therapies and resource uses observed in the JMDC database as follows and outcomes derived from the IORRA cohort were incorporated as a base case analysis.

### Cost

An overview of study drug costs, including those of ABA and weighted average costs of TNFi drugs, and concomitant drug cost inputs are provided in S5 and S6 Tables, respectively. Specific data on the regimens, including number of doses, administrations and treatment durations, are provided in S7 Table (S1 File). With regard to concomitant medication, prices per 1 mg were used in the model, as the dosage form used in the original model was different from the one used in Japan. Unit costs for serious adverse events (SAEs), including depression, urinary tract infections, gastroenteritis, bronchitis and pulmonary tuberculosis and malignancies, were obtained from the JMDC claims database as total costs per hospital admission (S8 Table). Cumulative incidence rates of SAEs were obtained from the IORRA database. Finally, costs for clinical assessment (disease monitoring costs), such as outpatient or inpatient follow-up visits, radiographic examinations and routine blood exams, were taken from the JMDC database (S9 Table).

Costs were not inflated in the base case but inflated to 2020 Japanese Yen (JPY) in a scenario analysis [16]. Unit prices of all medical services are officially decided at the different revision rates between expense item groups every 2 years in Japan. The correlation between the revision rates and consumer price indexes (CPIs) was positive in some expense item groups but negative or neutral in other expense item groups. Therefore, due to such complexity, we determined the unit prices of the included cost items for each year of use, although CPIs are usually used as the exchange rate to convert costs into those in the reported year. Hence, the inflation effect on costs was analysed in two scenarios, using CPI and the official revision rates per expense item groups, respectively.

### Outcomes

The primary endpoint was costs per (ACR50) responder, and secondary endpoints were costs per patient in Clinical Disease Activity Index (CDAI) and Simplified Disease Activity Index (SDAI) remission. There is no established methodology to statistically compare the calculated costs between the two groups; thus, the comparison was made according to previous studies. In one previous study, the magnitude of the cost was compared by using the ratio against the total cost [8], and in another study, lower costs by 25% and lower costs by 40% were used to show the difference in cost [17]. This study used the latter method and applied more stringent criteria (± 10%) to assess whether the cost was considered equivalent between the groups. Other outcomes of interest were incremental costs per additional responding patient or patient in remission, incremental cost per discontinued patient avoided and per SAE.

### Sensitivity analyses

One-way sensitivity analysis (OWSA) where input parameters were varied by ±30% was performed to assess the impact of model inputs on the results for total incremental costs. In addition, total incremental costs per health gain for ABA-1L were compared to ±10% of those for comparators. Finally, scenario analyses were conducted to determine the impact of age (<65 versus ≥65), disease duration (<5 years versus ≥5 years) and severity on treatment initiation (J-HAQ <1.5 versus J-HAQ ≥1.5), respectively.

## Results

### ABA-1L versus TNFi-1L

Out of the 1,000 patients in the initial hypothetical cohort, the number of ACR50 responders was 134 in both the ABA-1L and TNFi-1L arms. The number of patients in CDAI and SDAI remission in TNFi-1L (268 and 280, respectively) was slightly higher compared with that in ABA-1L (256 and 244, respectively) (Table 3). In addition, more patients discontinued treatment with TNFi-1L compared to ABA-1L (427 versus 256), and patients on ABA-1L experienced more SAEs than patients on TNFi-1L (161 versus 151), respectively.

**Table 3:** Health outcomes per cohort for the overall AMPLE population after 2 years (ABA-1L vs. TNFi-1L).

	ABA-1L	TNFi-1L	Incremental number of patients achieving endpoint (ABA-1L - TNFi-1L)
**Total number of responding patients**			
ACR50	134.15	134.15	0.00
J-HAQ	182.93	182.93	0.00
**Total number of patients in remission**			
CDAI	256.10	268.29	-12.20
SDAI	243.90	280.49	-36.59
**Total number of patients discontinuing treatment**			
Any reason	256.10	426.83	-170.73
**Total number of patients with an adverse event**			
Serious adverse events	160.63	150.92	9.71

1L, first line; ABA, abatacept; ACR50, American College of Rheumatology response of at least 50% improvement; CDAI, Clinical Disease Activity Index; J-HAQ, Japanese version of Health Assessment Questionnaire; SDAI, Simplified Disease Activity Index; TNFi, tumour necrosis factor inhibitor.

Total costs were lower for ABA-1L compared with TNFi-1L, with a difference in costs amounting to -1,571 JPY (-14.7 USD) PMPM over the 2-year time horizon (Table 4). The cost per resource favoured ABA-1L over TNFi-1L, except for hospitalisations due to infections (incremental difference of 153 JPY [1.4 USD]). With regard to the costs per health gain, the incremental costs PMPM by ACR50 response (i.e. the primary endpoint), CDAI remission and SDAI remission (i.e. secondary endpoints) for ABA-1L versus TNFi-1L were -11,715 JPY (-110 USD), 11,602 JPY (109 USD) and 47,003 JPY (440 USD), respectively (Table 5). Incremental costs per additional patient in CDAI remission and with ACR50 response for ABA-1L were within ±10% of those for TNFi-1L; however, incremental costs per additional patient in SDAI remission for ABA-1L were slightly higher than +10% of those for TNFi-1L.

**Table 4:** Cost results breakdown–per patient per month (ABA-1L vs. TNFi-1L).

	ABA-1L	TNFi-1L	Incremental costs (ABA-1L –TNFi-1L)
**Total costs per PMPM**			
bDMARD	73,057	74,513	-1,456
Concomitant medication	2,151	2,163	-12
Monitoring	19,376	19,376	0
AEs occurring ≥5%	0	0	0
SAEs	3,173	3,430	-257
Hospitalisations due to infections	612	459	153
Total	98,369	99,940	-1,571

1L, first line; ABA, abatacept; AE, adverse event; bDMARD, biological disease-modifying antirheumatic drug; PMPM, per member per month; SAE, serious AE; TNFi, tumour necrosis factor inhibitor.

**Table 5:** Cost per responder and patient in remission–per member per month (ABA-1L vs. TNFi-1L).

	Cost per health gain^a^ (ABA-1L)	Cost per health gain^a^ (TNFi-1L)	±10% incremental cost for TNFi-1L^b^	Difference in costs (ABA-1L –TNFi-1L)
Primary endpoint: cost per responding patient (JPY)				
ACR50	733,293	745,008	670,507; 819,509	-11,715
Secondary endpoints: cost per patient in remission (JPY)				
CDAI	384,106	372,504	335,254; 409,754	11,602
SDAI	403,311	356,308	320,677; 391,939	47,003

1L, first line; ABA, abatacept; ACR50, American College of Rheumatology response of at least 50% improvement; CDAI, Clinical Disease Activity Index; SDAI, Simplified Disease Activity Index; TNFi, tumour necrosis factor inhibitor.

^a^Total costs in ABA-1L or TNFi-1L divided by the number of ACR responders or patients in CDAI or SDAI remission.

^b^Crude sensitivity analysis where the incremental costs for TNFi-1L were increased/decreased by 10%.

Furthermore, the incremental cost PMPM for TNFi-1L per SAE avoided was 162 JPY (1.5 USD) and per additional patient in remission (CDAI and SDAI) 129 JPY (1.2 USD) and 43 JPY (0.4 USD), respectively. The decreasing cost for ABA-1L per discontinuation avoided was 9 JPY (0.1 USD), i.e. fewer patients discontinued with less costs.

The results of the OWSA showed that the unit costs of the two treatments (i.e. ABA-1L and TNFi-1L) were the most influential parameters on the difference in total costs (S10 Table). The duration and dosage of concomitant medications were the next most influential parameters, but the incremental costs were very small in comparison to the cost of treatments.

**Fig 3:**
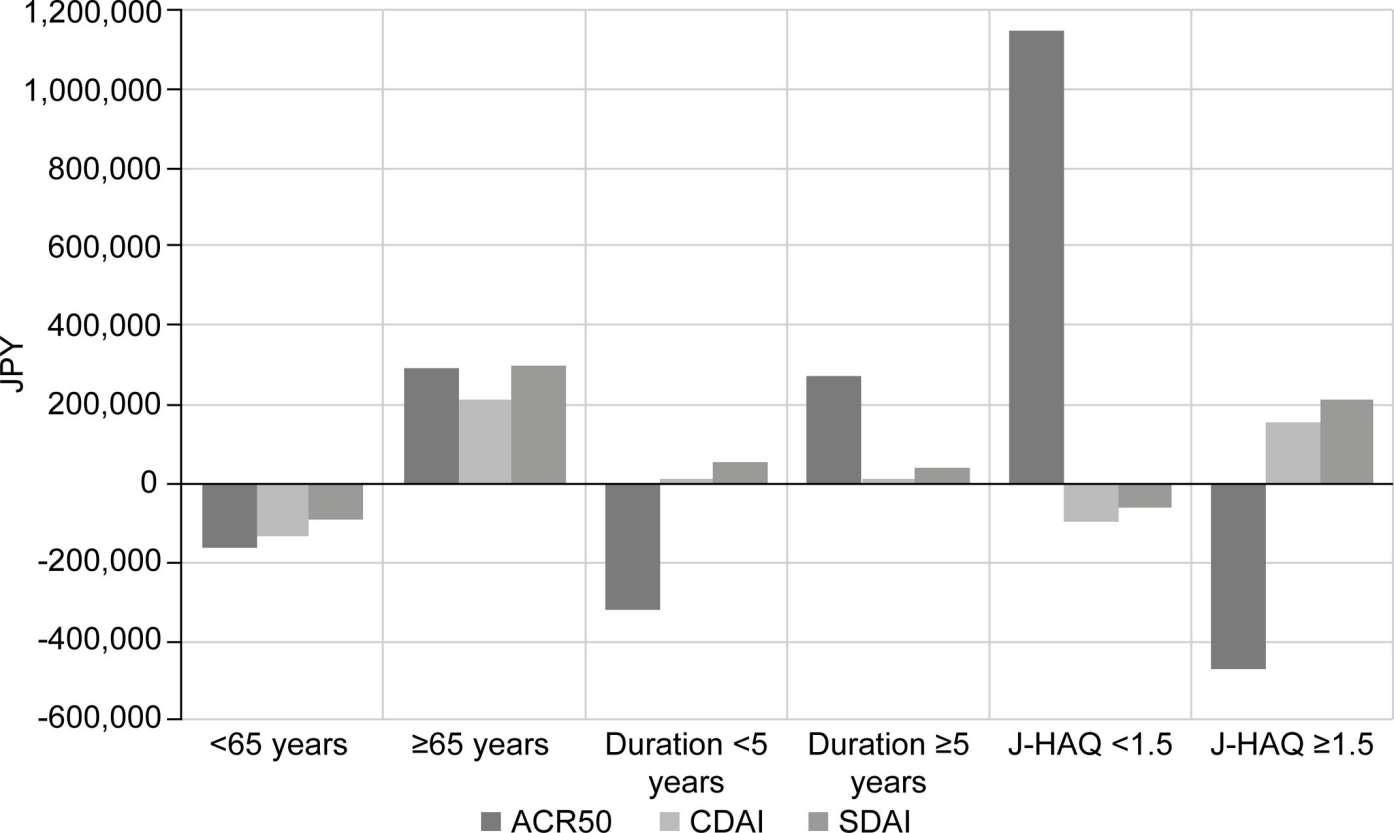
Scenario analyses: Differences in cost per patient achieving endpoints (ABA-1L vs. TNFi-1L). Difference in costs per health gain PMPM (ABA-1L –TNFi-1L). Costs per health gain PMPM were calculated by dividing total costs by the number of responders, patients in CDAI or SDAI remission for each group. The more positive the vertical axis, the more cost-effective the TNFi-1L was. 1L, first line; ABA, abatacept; ACR50, American College of Rheumatology response of at least 50% improvement; CDAI, Clinical Disease Activity Index; J-HAQ, Japanese version of Health Assessment Questionnaire; JPY, Japanese Yen; PMPM, per member per month; SDAI, Simplified Disease Activity Index; TNFi, tumour necrosis factor inhibitor.

For responding patients, with J-HAQ <1.5 assessed by ACR50, TNFi-1L was a cost-effective treatment option (incremental cost of 1.15 MJPY [10,769 USD] PMPM for ABA-1L), but no benefits were seen for patients in CDAI or SDAI remission (Fig 3). The reverse was observed for responding patients with J-HAQ ≥1.5 where ABA-1L was cost-effective (incremental cost of -0.47 MJPY [-425 USD] PMPM for ABA-1L) for patients achieving ACR50, while TNFi-1L was cost-effective for patients in CDAI or SDAI remission. For patients with a disease duration <5 years, ABA-1L was cost-effective for responding ACR50 patients (incremental costs approximately -0.3 MJPY [-3,021 USD] PMPM for ABA-1L), but a treatment preference was not seen for patients in CDAI or SDAI remission.

Lastly, neither of the adjustments of input costs based on CPI or the official revision rates had an impact on the interpretation of the results; the overall incremental costs PMPM changed from -1,571 JPY (-15 USD) in the base case to -1,573 JPY (-15 USD) and -1,574 JPY (–15 USD), respectively (Table 6).

**Table 6:** Cost results breakdown of the model in a hypothetical cohort of 1,000 patients–per member per month (incremental costs [ABA-1L vs. TNFi-1L]).

	Base case	CPI	Point rate	≥65 years	<65 years	Duration ≥5 years	Duration <5 years	J-HAQ <1.5	J-HAQ ≥1.5
bDMARD	-1,456	-1,456	-1,456	-1,456	-1,456	-1,456	-1,456	-1,456	-1,456
Concomitant medication	-12	-12	-12	-12	-12	-12	-12	-12	-12
Monitoring	0	0	0	0	0	0	0	0	0
AEs occurring ≥5%	0	0	0	0	0	0	0	0	0
SAEs	-257	-263	-261	129	-1,042	-347	28	-477	-78
Hospitalisations due to infections	153	157	154	153	153	153	153	153	153
Total	-1,571	-1,573	-1,574	-1,185	-2,357	-1,661	-1,287	-1,792	-1,392

1L, first line; ABA, abatacept; AE, adverse event; bDMARD, biological disease-modifying antirheumatic drug; CPI, consumer price index; J-HAQ, Japanese version of Health Assessment Questionnaire; Point rate, official revision rates per expense item; SAE, serious AE; TNFi, tumour necrosis factor inhibitor.

### ABA-1L versus ABA-2L+

When comparing ABA-1L and ABA-2L+ in the hypothetical cohort, the number of ACR50 responders in ABA-1L was higher than that in ABA-2L+ (113 and 99, respectively). The number of patients in both CDAI and SDAI remission in ABA-1L (268 and 268, respectively) was higher compared with that in ABA-2L+ (155 and 155, respectively) (Table 7). More patients discontinued treatment with ABA-2L+ compared with ABA-1L (437 versus 268, respectively), and patients on ABA-2L+ also experienced more SAEs than patients on ABA-1L (250 versus 234, respectively).

**Table 7:** Health outcomes per cohort for the overall AMPLE population after 2 years (ABA-1L vs. ABA-2L+).

	ABA-1L	ABA-2L+	Incremental number of patients achieving endpoint (ABA-1L –ABA-2L+)
**Total number of responding patients**			
ACR50	112.68	98.59	14.08
J-HAQ	183.10	112.68	70.42
**Total number of patients in remission**			
CDAI	267.61	154.93	112.68
SDAI	267.61	154.93	112.68
**Total number of patients discontinuing treatment**			
Any reason	267.61	436.62	-169.01
**Total number of patients with an adverse event**			
Serious adverse events	234.41	250.28	-15.87

1L, first line; 2L+, second or later line; ABA, abatacept; ACR50, American College of Rheumatology response of at least 50% improvement; CDAI, Clinical Disease Activity Index; J-HAQ, Japanese version of Health Assessment Questionnaire; SDAI, Simplified Disease Activity Index.

The overall additional cost of using ABA-1L compared with ABA-2L+ equalled 81 JPY (0.8 USD) PMPM over the 2-year time horizon, and the cost per outcome showed similar results for all resources, except for the cost for bDMARDs that was -435 JPY (-4.1 USD) PMPM (Table 8). Across primary and secondary endpoints, costs per health gain were lower in ABA-1L, with the incremental costs per ACR50 responder, CDAI remission and SDAI remission for ABA-1L versus ABA-2L+ being -122,824 JPY (-1,150 USD), -264,514 JPY (-2,477 USD) and -264,514 JPY (-2,477 USD), respectively (Table 9). Incremental costs per additional patient in CDAI and SDAI remission and with ACR50 response for ABA-1L were lower than -10% of those for ABA-2L+.

**Table 8:** Cost results breakdown–per member per month (ABA-1L vs. ABA-2L+).

	ABA-1L	Abatacept 2L+	Incremental costs (ABA-1L –ABA-2L+)
**Total costs per PMPM**			
bDMARD	71,722	72,156	-435
Concomitant medication	2,153	1,966	187
Monitoring	19,489	19,489	0
AEs occurring ≥5%	21	0	21
SAEs	3,699	3,446	253
Hospitalisations due to infections	439	384	55
Total	97,522	97,441	81

1L, first line; 2L+, second or later line; ABA, abatacept; AE, adverse event; bDMARD, biological disease-modifying antirheumatic drug; PMPM, per member per month; SAE, serious AE.

**Table 9:** Cost per responder and patient in remission–per member per month (ABA-1L vs. ABA-2L+).

	Cost per health gain^a^ (ABA-1L)	Cost per health gain^a^ (ABA-2L+)	±10% incremental cost for ABA-2L+^b^	Difference in costs (ABA-1L - ABA-2L+)
Primary endpoint: cost per responding patient (JPY)				
ACR50	865,509	988,333	889,500; 1,087,166	-122,824
Secondary endpoints: cost per patient in remission (JPY)				
CDAI	364,425	628,939	566,045; 691,833	-264,514
SDAI	364,425	628,939	566,045; 691,833	-264,514

1L, first line; 2L+, second or later line; ABA, abatacept; ACR50, American College of Rheumatology response of at least 50% improvement; CDAI, Clinical Disease Activity Index; SDAI, Simplified Disease Activity Index.

^a^Total costs in ABA-1L or ABA-2L+ divided by the number of ACR responders or patients in CDAI or SDAI remission.

^b^Crude sensitivity analysis where the incremental costs for ABA-2L+ were increased/decreased by 10%.

The cost for SAE was higher (253 JPY or 2.4 USD PMPM) for ABA-1L compared with ABA-2L+ after 2 years, with ABA-1L patients experiencing bronchitis, gastroenteritis or pulmonary tuberculosis (Table 8). The incremental cost PMPM for ABA-1L per SAE avoided was 5 JPY (0.05 USD), per additional ACR50 responder 6 JPY (0.05 USD) and per additional patient in remission (CDAI and SDAI) 1 JPY (0.01 USD), respectively. Finally, the incremental cost per discontinuation avoided was 0 JPY (0.00 USD).

Furthermore, the OWSA showed that the weekly unit costs for ABA-1L and ABA-2L+ were the most influential parameters on the difference in total costs (S11 Table).

**Fig 4:**
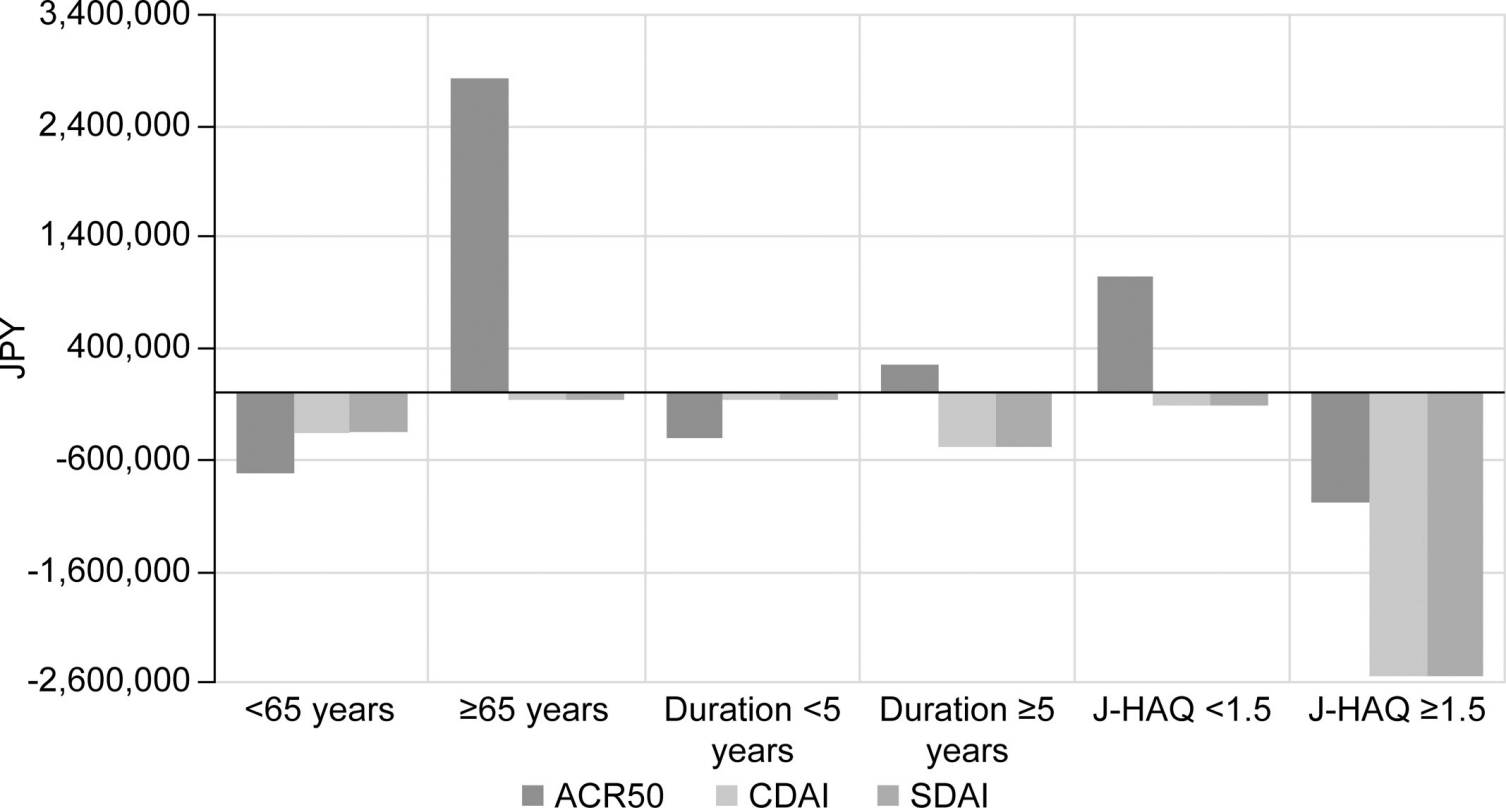
Scenario analyses: Differences in cost per patient achieving endpoints (ABA-1L vs. ABA-2L+). Difference in costs per health gain PMPM (ABA-1L –ABA-2L+). Costs per health gain PMPM were calculated by dividing total costs by the number of responders, patients in CDAI or SDAI remission for each group. The more positive the vertical axis, the more cost-effective the ABA-2L+ was. 1L, first line; 2L+, second or later line; ABA, abatacept; ACR50, American College of Rheumatology response of at least 50% improvement; CDAI, Clinical Disease Activity Index; J-HAQ, Japanese version of Health Assessment Questionnaire; JPY, Japanese Yen; PMPM, per member per month; SDAI, Simplified Disease Activity Index.

For the subgroup of patients <65 years or with J-HAQ ≥1.5, the results were robust, and ABA-1L was the cost-efficient treatment regardless of the use of ACR50, CDAI or SDAI (Fig 4). However, for patients ≥65 years, ABA-2L+ was more cost-effective than ABA-1L (incremental cost of 2.8 MJPY [26,474 USD] PMPM for ABA-1L) for responding patients measured with ACR50, while cost benefits were similar between ABA-1L and ABA-2L+ for patients in CDAI or SDAI remission. For patients with a disease duration ≥5 years, ABA-2L+ was cost-effective for responding patients (incremental cost of 0.26 MJPY [2,463 USD] PMPM for ABA-1L) measured with ACR50, while ABA-1L was preferred for patients in CDAI or SDAI remission (incremental cost of -0.48 MJPY [-4,453 USD] PMPM for ABA-1L).

Furthermore, the incremental cost for SAE (ABA-1L - ABA-2L+) was higher for patients ≥65 years compared with the base case; it was 722 JPY (7 USD) in patients ≥65 years versus 253 JPY (2 USD) in the base case PMPM, respectively. The opposite was true for patients <65 years with an incremental cost of -419 JPY (-4 USD) versus 253 JPY (2 USD) in the base case (Table 10).

**Table 10:** Cost results breakdown of the model in a hypothetical cohort of 1,000 patients–per member per month (Incremental costs [ABA-1L vs. TNFi-1L]).

	Base case	CPI	Point rate	≥65 years	<65 years	Duration ≥5 years	Duration <5 years	J-HAQ <1.5	J-HAQ ≥1.5
bDMARD	-435	-435	-435	-435	-435	-435	-435	-435	-435
Concomitant medication	187	187	187	187	187	187	187	187	187
Monitoring	0	0	0	0	0	0	0	0	0
Aes occurring ≥5%	21	21	21	0	42	27	0	0	45
SAEs	253	259	242	722	-419	45	102	589	8
Hospitalisations due to infections	55	56	55	55	55	55	55	55	55
Total	81	88	71	529	-570	-121	-91	396	-140

1L, first line; ABA, abatacept; AE, adverse event; bDMARD, biological disease-modifying antirheumatic drug; CPI, consumer price index; J-HAQ, Japanese version of Health Assessment Questionnaire; Point rate, official revision rates per expense item; SAE, serious AE; TNFi, tumour necrosis factor inhibitor.

Similar to the comparison between ABA-1L and TNFi-1L, neither of the adjustments of input costs based on CPI or the official revision rates had an impact on the interpretation of the results, and the overall incremental costs changed from 81 JPY (0.8 USD) in the base case to 88 JPY (0.8 USD) and 71 JPY (0.7 USD), respectively.

## Discussion

In the present study, we found that there was no difference (i.e. within ±10%) in cost-effectiveness between ABA-1L and TNFi-1L per ACR50 responder and per patient in CDAI remission and a marginal difference (i.e. +13%) per patient in SDAI remission, although the total cost was lower for ABA-1L compared to TNFi-1L over the 2-year time horizon. Costs per health gain were more than 10% lower in ABA-1L versus ABA-2L+ with incremental costs per ACR50 responder, CDAI and SDAI remission, although the total cost showed similar results for ABA-2L+ and ABA-1L. In both comparisons, the cost of treatment (e.g. bDMARD) had the largest impact on the difference in total costs.

We have previously shown the long-term cost-effectiveness of tocilizumab compared with TNFi [8]; however, in the current study, unlike the previous study, we have presented the short-term cost-effectiveness focusing on ABA. Drug selection for elderly RA patients is a challenge in an ageing society. As ABA is relatively often used in the elderly from the viewpoint of safety [11], the current study to determine its cost-effectiveness is of significance. We have not performed stratified analyses in the current study, and future research is warranted to examine the cost-effectiveness in high-risk patient groups in an ageing society. The cost-effectiveness of ABA-1L was shown to be similar to that of TNFi-1L, indicating that, from a health economics point of view, ABA can be used as the 1L treatment in patients with inadequate response to MTX or csDMARDs. On the other hand, the effectiveness of bDMARDs as the 2L+ treatment generally tends to decrease, and these findings are not limited to ABA [12, 18]; therefore, the cost-effectiveness of the 2L+ treatment is expected to be lower than that of the 1L treatment for any bDMARD, and the cost-effectiveness of ABA-2L+ treatment in the current study is reasonable. However, in clinical practice, ABA tends to be used in high-risk patients and difficult to treat RA such as those with an inadequate response to csDMARDs or bDMARDs [19, 20]. It is important to select the most appropriate 1L drug for each patient not only from the viewpoint of effectiveness and safety but also from the health economics perspective. Several head-to-head cost-effectiveness studies of bDMARDs have been reported [6, 21–25]. Reports of ABA-1L versus adalimumab-1L in bDMARD-naïve RA patients indicated that ABA-1L was cost-effective, especially in anti-citrullinated protein antibody (ACPA)-positive patients [6, 21]. Reports of rituximab-1L [22] or tocilizumab-1L [24] versus TNFi-1L in bDMARD-naïve RA patients indicated that rituximab-1L or tocilizumab-1L was cost-effective. However, the direct comparison of the cost-effectiveness of ABA-1L and TNFi-1L has not been reported. In addition, there have been no reports regarding the cost-effectiveness of ABA-1L and ABA-2L+. These cost-effectiveness analyses were examined for the first time in this study.

In our recent study [8], introduction of bDMARDs was shown to be a cost-effective treatment for RA patients in Japan, especially for the subgroup of patients <50 years with J-HAQ between 1.1 and 1.6. Similar results were demonstrated in this cost-effectiveness study where ABA-1L was more cost-effective against ABA-2L+ in a comparable subgroup of patients (patients <65 years or J-HAQ ≥1.5). The presented result highlights the importance of considering the subgroup and response to a treatment when evaluating the cost-effectiveness of the investigated treatments.

The major strength of this study is the use of real-world data derived from large, well-established and comprehensive databases, which renders the results easily translatable to clinical settings in Japan, thus facilitating RA treatment decision-making. This is also the first study to demonstrate the cost-effectiveness of ABA-1L against TNFi-1L as well as against ABA-2L+ for selected subgroups of patients (responding patients <65 years). We have performed different scenario analyses using several outcomes, among which the scenario showing a consistent trend in all outcomes in the differences in cost per patient achieving endpoints is deemed more robust than the scenario that showed different trends.

Several limitations of this study should be noted. The short time horizon (i.e., 2 years) is a limitation, considering the chronic and progressive nature of the disease. However, extending the time horizon would require either longitudinal data from the trial, which are not available, or simplifying assumptions for subsequent treatment sequences, which is impractical, given the various therapy options that are possible in RA. Rather than extending the analysis beyond the available AMPLE data by applying assumptions, it was preferred to perform a more robust analysis for Japan, relying only on data from the trial and inputs from the IORRA database. Moreover, as a limited number of patients were prescribed ABA-1L treatment in the JMDC database, we could not account for AEs with low incidence rate and assess their respective cost, which means that actual AE management costs in ABA-1L are likely to be higher. In addition, since disease activity scores were not collected in the JMDC database, no matching on disease severity between patient groups could be conducted. Unadjusted disease severity between groups is likely to cause the difference in incidence rate and level of health resource utilisation. Furthermore, although the cost-effectiveness of ABA has been demonstrated in ACPA-positive patients [5, 21], in this study it was not possible to investigate by ACPA status or with/without MTX in relation to the number of patients. The fact that there were many patient data unused, especially in the TNFi-1L group, as matching was performed to enable comparison between treatments could also be considered as a limitation.

Finally, the analysis did not incorporate the impact of health-related quality of life (HRQoL). In the present study, however, the feasibility of applying HRQoL was challenged by the lack of utility data. In the future, re-running the analysis by considering utility data could provide a sound basis for health policy initiatives as cost-utility analysis is often the preferred analytical approach of decision-makers requiring health economic evidence. In addition, analysis of data stratified by ACPA status and/or by use of MTX would provide valuable insights and deeper understanding on the relation between the economic attributes of the early use of ABA and patients’ characteristics.

## Conclusions

There was no or marginal difference in the cost-effectiveness between ABA-1L and TNFi-1L in RA patients; however, ABA-1L was found to be cost-saving against ABA-2L+ and will accrue savings for the Japanese healthcare payers.

## Supporting information

S1 File Supplementary methods. (DOCX)

S1 Table Eligible patient population from the JMDC claims database (ABA-1L vs. TNFi-1L). ^a^Paired t-tests were used. ^b^Disease duration (from date of initial diagnosis to ABA/TNFi start date). 1L, first line; ABA, abatacept; df, degrees of freedom; JMDC, Japan Medical Data Center Inc; SD, standard deviation; TNFi, tumour necrosis factor inhibitor.
(DOCX)

S2 Table Eligible patient population from the JMDC claims database (ABA-1L vs. ABA-2L+). 1L, first line; 2L+, second or later line; ABA, abatacept; df, degrees of freedom; JMDC, Japan Medical Data Center Inc; SD, standard deviation. ^a^McNemar’s chi-squared test or paired t-tests were used. ^b^Disease duration (from date of initial diagnosis to ABA start date).
(DOCX)

S3 Table Effectiveness from the IORRA database (ABA-1L vs. TNFi-1L). 1L, first line; ABA, abatacept; ACR50, American College of Rheumatology response of at least 50% improvement; CDAI, Clinical Disease Activity Index; IORRA, Institute of Rheumatology, Rheumatoid Arthritis; J-HAQ, Japanese version of Health Assessment Questionnaire; SDAI, Simplified Disease Activity Index; TNFi, tumour necrosis factor inhibitor.
(DOCX)

S4 Table Effectiveness from the IORRA database (ABA-1L *vs*. ABA-2L+). 1L, first line; 2L+, second or later line; ABA, abatacept; ACR50, American College of Rheumatology response of at least 50% improvement; CDAI, Clinical Disease Activity Index; IORRA, Institute of Rheumatology, Rheumatoid Arthritis; J-HAQ, Japanese version of Health Assessment Questionnaire; SDAI, Simplified Disease Activity Index.
(DOCX)

S5 Table Weighted average cost of abatacept and TNFi drugs. JMDC, Japan Medical Data Center Inc; JPY, Japanese Yen; mg, milligram; MTX, methotrexate; TNFi, tumour necrosis factor inhibitor.
(DOCX)

S6 Table Concomitant drug costs. Source: JMDC Claims Database. JMDC, Japan Medical Data Center Inc; JPY, Japanese Yen; mg, milligram.
(DOCX)

S7 Table Study dosages, administration and treatment duration. 1L, first line; 2L+ second or later line; ABA, abatacept; IORRA, Institute of Rheumatology, Rheumatoid Arthritis; JMDC, Japan Medical Data Center Inc; mg, milligram; MTX, methotrexate; TNFi, tumour necrosis factor inhibitor. ^a^Value used: ABA-1L vs. TNFi-1L/ABA-1L vs. ABA-2L+. ^b^Weighted average of etanercept 25/50 mg; adalimumab 20/40/80 mg; golimumab 50 mg; certolizumab pegol 200 mg from the JMDC database.
(DOCX)

S8 Table Adverse event costs. Source: JMDC Claims Database. 1L, first line; 2L+, second or later line; ABA, abatacept; JPY, Japanese Yen; TNFi, tumour necrosis factor inhibitor.
(DOCX)

S9 Table Disease monitoring costs. Source: JMDC Claims Database. 1L, first line; 2L+, second or later line; ABA, abatacept; JPY, Japanese Yen; TNFi, tumour necrosis factor inhibitor.
(DOCX)

S10 Table One-way sensitivity analysis: ABA-1L vs. TNFi-1L. Ten most influential parameters on the difference in total costs (JPY). Minimum: -719,173, 388; Maximum: 1,040,202,718; Base case: -37,715,332. 1L, first line; ABA, abatacept; MTX, methotrexate; NSAID, non-steroidal anti-inflammatory drug; TNFi-, tumour necrosis factor inhibitor.
(DOCX)

S11 Table One-way sensitivity analysis: ABA 1L vs. ABA 2L+. Ten most influential parameters on the difference in total costs (JPY). Minimum: -719,173, 388; Maximum: 1,040,202,718; Base case: 1,941,292. 1L, first line; 2L+, second or later line; ABA, abatacept; MTX, methotrexate; NSAID, non-steroidal anti-inflammatory drug.
(DOCX)
