# Supplementary material for: Cost-consequence of abatacept as first-line therapy in Japanese rheumatoid arthritis patients using IORRA real-world data
Source: PLoS One. 2022 Nov 16;17(11):e0277566. doi: 10.1371/journal.pone.0277566 (PMC9668164; doi:10.1371/journal.pone.0277566)
Supplement: S1 File — (DOCX) [file pone.0277566.s001.docx]

**Supplementary methods**

***Outcomes***

Effectiveness outcomes (percentage of ACR50 responders, those who achieved CDAI remission and those who achieved SDAI remission) were calculated for 2 years after the initiation of ABA or TNFi in a population of patients in the matched IORRA cohort. The percentage of ACR 50 responders or those who achieved CDAI or SDAI remission at the last observation in the 2 years was calculated, including patients who could not be observed for 2 years due to drug discontinuation or withdrawal. The Kaplan-Meier method was used to calculate the proportion of patients who had ≥1 adverse events during the observation period of up to 2 years. We also calculated the outcomes and incidence rates of adverse events for each subgroup for scenario analysis (S4 Table).

***Costs***

Some costs were derived from the JMDC database. The patient inclusion criteria for cost calculation were as follows: ABA or TNFi prescriptions between 1 September 2010 and 30 September 2019 (first prescription date was the index date); medical record of rheumatoid arthritis (ICD-10: M05.x, M06.x, except M06.1) even once in 6 months before the index date; no medical record for adult still disease (ICD-10: M06.1) or juvenile rheumatoid arthritis (ICD-10: M08.x) within 6 months before index date; observable longer than 6 months before index date and (first-line group only) no other molecular target drug prescriptions within 6 months before index date. Furthermore, a random sampling was applied so that the distribution of sex, age and disease duration was equivalent to that in the patient population of the IORRA cohort after matching. As a result, 230 patients each for ABA-1L versus TNFi‑1L and 144 patients each for ABA-1L versus ABA-2L+ were used for resource utilization calculation. Adverse events that could not be confirmed to occur in the JMDC‑derived population were excluded from the analysis in this study because resource utilization for treatment could not be calculated.

**Statistical analyses**

Patient matching was performed to allow comparison between ABA-1L versus TNFi-1L and ABA-1L versus ABA-2L+. For the former, propensity score matching was performed using logistic regression analysis, with sex, age, J-HAQ, DAS28, disease duration (years), MTX dose, presence or absence of MTX use and presence or absence of comorbidities considered as variables. The accuracy of matching was evaluated by C statistic and standardization difference. For the latter, an exact matching was performed under the conditions of same sex, age ± 5 years, DAS28 ± 1.0 and disease duration ± 5 years. Assuming that the distribution difference of each variable between the two groups before matching remains after matching, the minimum number of patients required to detect the distribution difference between the two groups was 970 for ABA-1L versus TNFi-1L in the IORRA cohort, 40 for ABA-1L versus TNFi-1L in the JMDC-derived population, 40 for ABA-1L versus ABA-2L+ in the IORRA cohort and 10 for ABA-1L versus ABA-2L+ in the JMDC-derived population, with a significance level of 5% and a power of 70%. The power was first deemed insufficient for ABA-1L versus TNFi-1L in the IORRA cohort considering the number of patients after matching, but after matching using propensity scores, the standardization difference was less than 0.2 except for type 2 diabetes and the C statistic was 0.79. Hence, it was judged that matching re-examination was not necessary.

Patient characteristics for ABA-1L versus TNFi-1L and ABA-1L versus ABA-2L+ were statistically compared in the IORRA cohort before and after matching. Similarly, patient characteristics before and after sampling were statistically compared for the JMDC-derived patient population that was randomly sampled so that the distribution of sex, age and disease duration were equivalent to that in the IORRA cohort. No statistical evaluation was performed on the effectiveness and cost estimation results using the model. All statistical analyses were performed using R (3.9.0).
