# Supplementary material for: Cost-consequence of abatacept as first-line therapy in Japanese rheumatoid arthritis patients using IORRA real-world data
Source: PLoS One. 2022 Nov 16;17(11):e0277566. doi: 10.1371/journal.pone.0277566 (PMC9668164; doi:10.1371/journal.pone.0277566)
Supplement: S1 Table — aPaired t-tests were used. bDisease duration (from date of initial diagnosis to ABA/TNFi start date). 1L, first line; ABA, abatacept; df, degrees of freedom; JMDC, Japan Medical Data Center Inc; SD, standard deviation; TNFi, tumour necrosis factor inhibitor. (DOCX) [file pone.0277566.s002.docx]

**S1 Table. Eligible patient population from the JMDC claims database (ABA-1L vs. TNFi-1L).**

|  | ABA-1L | | TNFi-1L | | Statistics | | |
| --- | --- | --- | --- | --- | --- | --- | --- |
|  | N or mean | % or SD | N or mean | % or SD | t^a^ or χ^2^ value | df | p value |
| N | 230 |  | 230 |  |  |  |  |
| Sex (female) | 184 | 80.0% | 184 | 80.0% | 0.00 | 1.00 | 1.0000 |
| Age | 54.73 | 10.94 | 54.13 | 10.83 | 0.47 | 229 | 0.6353 |
| Disease duration (yrs)^b^ | 2.17 | 1.74 | 2.04 | 1.80 | 2.60 | 229 | 0.0100 |

^a^Paired t-tests were used.

^b^Disease duration (from date of initial diagnosis to ABA/TNFi start date).

1L, first line; ABA, abatacept; df, degrees of freedom; JMDC, Japan Medical Data Center Inc; SD, standard deviation; TNFi, tumour necrosis factor inhibitor.
