# Supplementary material for: Cost-consequence of abatacept as first-line therapy in Japanese rheumatoid arthritis patients using IORRA real-world data
Source: PLoS One. 2022 Nov 16;17(11):e0277566. doi: 10.1371/journal.pone.0277566 (PMC9668164; doi:10.1371/journal.pone.0277566)
Supplement: S2 Table — 1L, first line; 2L+, second or later line; ABA, abatacept; df, degrees of freedom; JMDC, Japan Medical Data Center Inc; SD, standard deviation. aMcNemar’s chi-squared test or paired t-tests were used. bDisease duration (from date of initial diagnosis to ABA start date). (DOCX) [file pone.0277566.s003.docx]

**S2 Table. Eligible patient population from the JMDC claims database (ABA-1L vs. ABA-2L).**

|  | ABA-1L | | ABA-2L | |  | Statistics |  |
| --- | --- | --- | --- | --- | --- | --- | --- |
|  | N or mean | % or SD | N or mean | % or SD | t^a^ or χ^2^ value | df | p value |
| N | 144 |  | 144 |  |  |  |  |
| Gender (female) | 135 | 93.8% | 135 | 93.8% | 0.00 | 1.00 | 1.0000 |
| Age | 54.20 | 9.64 | 53.93 | 9.51 | 0.22 | 143 | 0.8284 |
| Disease duration (yrs)^b^ | 2.29 | 1.91 | 2.68 | 1.94 | 1.37 | 143 | 0.1726 |

1L, first line; 2L, second or higher line; ABA, abatacept; df, degrees of freedom; JMDC, Japan Medical Data Center Inc; SD, standard deviation.

^a^McNemar’s chi-squared test or paired t-tests were used.

^b^Disease duration (from date of initial diagnosis to ABA start date).
