# Supplementary material for: Cost-consequence of abatacept as first-line therapy in Japanese rheumatoid arthritis patients using IORRA real-world data
Source: PLoS One. 2022 Nov 16;17(11):e0277566. doi: 10.1371/journal.pone.0277566 (PMC9668164; doi:10.1371/journal.pone.0277566)
Supplement: S3 Table — 1L, first line; ABA, abatacept; ACR50, American College of Rheumatology response of at least 50% improvement; CDAI, Clinical Disease Activity Index; IORRA, Institute of Rheumatology, Rheumatoid Arthritis; J-HAQ, Japanese version of Health Assessment Questionnaire; SDAI, Simplified Disease Activity Index; TNFi, tumour necrosis factor inhibitor. (DOCX) [file pone.0277566.s004.docx]

**S3 Table. Effectiveness from the IORRA database (ABA-1L vs. TNFi-1L).**

|  | | ABA-1L | | | | TNFi-1L | | | |
| --- | --- | --- | --- | --- | --- | --- | --- | --- | --- |
|  |  | N | ACR50 response | CDAI  remission | SDAI  remission | N | ACR50 response | CDAI  remission | SDAI  remission |
| Base case | | 82 | 13.4% | 25.6% | 24.4% | 82 | 13.4% | 26.8% | 28.0% |
| Scenario 1 Age | ≥65 years | 44 | 9.1% | 18.2% | 15.9% | 40 | 12.5% | 30.0% | 30.0% |
|  | <65 years | 38 | 18.4% | 34.2% | 34.2% | 42 | 14.3% | 23.8% | 26.2% |
| Scenario 2 Disease duration | ≥5 years | 63 | 9.5% | 23.8% | 22.2% | 61 | 13.1% | 24.6% | 24.6% |
|  | <5 years | 19 | 26.3% | 31.6% | 31.6% | 21 | 14.3% | 33.3% | 38.1% |
| Scenario 3 J-HAQ at baseline | <1.5 | 40 | 5.6% | 33.3% | 33.3% | 43 | 16.3% | 25.6% | 27.9% |
|  | ≥1.5 | 42 | 19.6% | 19.6% | 17.4% | 39 | 10.3% | 28.2% | 28.2% |

1L, first line; ABA, abatacept; ACR50, American College of Rheumatology response of at least 50% improvement; CDAI, Clinical Disease Activity Index; IORRA, Institute of Rheumatology, Rheumatoid Arthritis; J-HAQ, Japanese version of Health Assessment Questionnaire; SDAI, Simpliﬁed Disease Activity Index; TNFi, tumour necrosis factor inhibitor.
