# Supplementary material for: Cost-consequence of abatacept as first-line therapy in Japanese rheumatoid arthritis patients using IORRA real-world data
Source: PLoS One. 2022 Nov 16;17(11):e0277566. doi: 10.1371/journal.pone.0277566 (PMC9668164; doi:10.1371/journal.pone.0277566)
Supplement: S4 Table — 1L, first line; 2L+, second or later line; ABA, abatacept; ACR50, American College of Rheumatology response of at least 50% improvement; CDAI, Clinical Disease Activity Index; IORRA, Institute of Rheumatology, Rheumatoid Arthritis; J-HAQ, Japanese version of Health Assessment Questionnaire; SDAI, Simplified Disease Activity Index. (DOCX) [file pone.0277566.s005.docx]

**S4 Table. Effectiveness from the IORRA database (ABA-1L vs. ABA-2L+).**

|  | | ABA-1L | | | | ABA-2L+ | | | |
| --- | --- | --- | --- | --- | --- | --- | --- | --- | --- |
|  |  | N | ACR50 response | CDAI  remission | SDAI  remission | N | ACR50 response | CDAI  remission | SDAI  remission |
| Base case | | 71 | 11.3% | 26.8% | 26.8% | 82 | 9.9% | 15.5% | 15.5% |
| Scenario 1 Age | ≥65 years | 37 | 2.7% | 16.2% | 16.2% | 37 | 11.8% | 14.7% | 30.0% |
|  | <65 years | 34 | 20.6% | 38.2% | 38.2% | 34 | 8.1% | 16.2% | 26.2% |
| Scenario 2 Disease duration | ≥5 years | 54 | 7.4% | 24.1% | 24.1% | 54 | 9.3% | 11.1% | 24.6% |
|  | <5 years | 17 | 23.5% | 35.3% | 35.3% | 17 | 11.8% | 29.4% | 38.1% |
| Scenario 3 J-HAQ at baseline | <1.5 | 38 | 5.3% | 34.2% | 34.2% | 40 | 12.5% | 25.0% | 27.9% |
|  | ≥1.5 | 33 | 18.2% | 18.2% | 18.2% | 31 | 6.5% | 3.2% | 28.2% |

1L, first line; 2L+, second or later line; ABA, abatacept; ACR50, American College of Rheumatology response of at least 50% improvement; CDAI, Clinical Disease Activity Index; IORRA, Institute of Rheumatology, Rheumatoid Arthritis; J-HAQ, Japanese version of Health Assessment Questionnaire; SDAI, Simpliﬁed Disease Activity Index.
