# Supplementary material for: Cost-consequence of abatacept as first-line therapy in Japanese rheumatoid arthritis patients using IORRA real-world data
Source: PLoS One. 2022 Nov 16;17(11):e0277566. doi: 10.1371/journal.pone.0277566 (PMC9668164; doi:10.1371/journal.pone.0277566)
Supplement: S5 Table — JMDC, Japan Medical Data Center Inc; JPY, Japanese Yen; mg, milligram; MTX, methotrexate; TNFi, tumour necrosis factor inhibitor. (DOCX) [file pone.0277566.s006.docx]

**S5 Table. Weighted average cost of abatacept and TNFi drugs.**

| Drug | Unit price | Dose | Observed in JMDC database | | Estimated values | |
| --- | --- | --- | --- | --- | --- | --- |
|  |  |  | mg/week | Share per ingredient | Unit price weighted by share/week | Weighted average unit price/week |
| **Abatacept** |  |  |  |  |  |  |
| Abatacept 125 mg | 28,375 JPY | 125 mg/week | 125.1 | 100% | 28,375 | 28,375 JPY |
| **TNFi** |  |  |  |  |  |  |
| Etanercept 25 mg | 12,739 JPY | 25-50 mg/week | 31.5 | 35.70% | 5,722 | 26,267 JPY |
| Etanercept 50 mg | 25,317 JPY | 25-50 mg/week |  |  |  |  |
| Adalimumab 20 mg | 31,868 JPY | 40 mg/2 weeks |  |  |  |  |
| Adalimumab 40 mg | 62,976 JPY | 40 mg/2 weeks | 21.6 | 24.20% | 8,229 |  |
| Adalimumab 80 mg | 122,397 JPY | 40 mg/2 weeks |  |  |  |  |
| Golimumab 50 mg | 119,252 JPY | 50 mg/4 weeks (with MTX) | 13.3 | 25.50% | 8.065 |  |
| Certolizumab pegol 200 mg | 60,688 JPY | 400 mg/4 weeks | 95.6 | 14.60% | 4,251 |  |
|  |  |  |  |  |  |  |
| MTX | 210 JPY | 2.0 mg/week |  | 100% |  | 210 JPY |

JMDC, Japan Medical Data Center Inc; JPY, Japanese Yen; mg, milligram; MTX, methotrexate; TNFi, tumour necrosis factor inhibitor.
