# Supplementary material for: Cost-consequence of abatacept as first-line therapy in Japanese rheumatoid arthritis patients using IORRA real-world data
Source: PLoS One. 2022 Nov 16;17(11):e0277566. doi: 10.1371/journal.pone.0277566 (PMC9668164; doi:10.1371/journal.pone.0277566)
Supplement: S6 Table — Source: JMDC Claims Database. JMDC, Japan Medical Data Center Inc; JPY, Japanese Yen; mg, milligram. (DOCX) [file pone.0277566.s007.docx]

**S6 Table. Concomitant drug costs.**

| Regimen | Administration route | Unit | Cost (JPY) |
| --- | --- | --- | --- |
| Sulfasalazine (Azulfidine^®^) | Oral | 100 × 500 mg | 4,760 |
| Methylprednisolone/Prednisolone (Medrol^®^) | Oral | 4 mg | 15.9 |
| Non-steroidal anti-inflammatory drugs (Ibuprofen) | Oral | 200 mg | 8.1 |

Source: JMDC Claims Database.

JMDC, Japan Medical Data Center Inc; JPY, Japanese Yen; mg, milligram.
