# Supplementary material for: Cost-consequence of abatacept as first-line therapy in Japanese rheumatoid arthritis patients using IORRA real-world data
Source: PLoS One. 2022 Nov 16;17(11):e0277566. doi: 10.1371/journal.pone.0277566 (PMC9668164; doi:10.1371/journal.pone.0277566)
Supplement: S7 Table — 1L, first line; 2L+ second or later line; ABA, abatacept; IORRA, Institute of Rheumatology, Rheumatoid Arthritis; JMDC, Japan Medical Data Center Inc; mg, milligram; MTX, methotrexate; TNFi, tumour necrosis factor inhibitor. aValue used: ABA-1L vs. TNFi-1L/ABA-1L vs. ABA-2L+. bWeighted average of etanercept 25/50 mg; adalimumab 20/40/80 mg; golimumab 50 mg; certolizumab pegol 200 mg from the JMDC database. (DOCX) [file pone.0277566.s008.docx]

**S7 Table. Study dosages, administration and treatment duration.**

|  | ABA-1L^a^ | TNFi-1L | ABA-2L | Source |
| --- | --- | --- | --- | --- |
| Dose per week (mg) | 125/125 | ^b^ | 125 | Drug package insert |
| Mean number of missed injections at 24‑months | 3.30/3.30 | 3.30 | 3.30 | AMPLE trial^c^ |
| Administrations per month | 4.35/4.35 | 4.35 | 4.35 | Weekly injections. Calculated as: 365/12/7=4.35. |
| Mean duration (months) | 14.96/14.70 | 16.41 | 14.79 | Calculated from IORRA database |
| MTX mean weekly dose at 24-months (mg) | 6.39/6.75 | 6.08 | 6.50 | JMDC Claims Database |

1L, first line; 2L+, second or later line; ABA, abatacept; IORRA, Institute of Rheumatology, Rheumatoid Arthritis; JMDC, Japan Medical Data Center Inc; mg, milligram; MTX, methotrexate; TNFi, tumour necrosis factor inhibitor.

^a^Value used: ABA-1L vs. TNFi-1L/ABA-1L vs. ABA-2L.

^b^Weighted average of etanercept 25/50 mg; adalimumab 20/40/80 mg; golimumab 50 mg; certolizumab pegol 200 mg from the JMDC database.

^c^Source: IM101235. No authors listed. A randomized, head-to-head, single-blind study to compare the efficacy and safety of subcutaneous abatacept versus subcutaneous adalimumab, both with background methotrexate, in biologic-naive subjects with rheumatoid arthritis. Data on file.
