# Supplementary material for: Cost-consequence of abatacept as first-line therapy in Japanese rheumatoid arthritis patients using IORRA real-world data
Source: PLoS One. 2022 Nov 16;17(11):e0277566. doi: 10.1371/journal.pone.0277566 (PMC9668164; doi:10.1371/journal.pone.0277566)
Supplement: S8 Table — Source: JMDC Claims Database. 1L, first line; 2L+, second or later line; ABA, abatacept; JPY, Japanese Yen; TNFi, tumour necrosis factor inhibitor. (DOCX) [file pone.0277566.s009.docx]

**S8 Table. Adverse event costs.**

| Event |  | Unit | Cost (JPY)  ABA-1L vs. ABA-2L+ | Cost (JPY)  ABA-1L vs. TNFi-1L |
| --- | --- | --- | --- | --- |
| Depression |  | Per day hospital | 32,455.55 | 21,530.31 |
| Urinary tract infection |  | Per admission (hospitalisation > 1 day) | 265,934.97 | 781,846.48 |
| Gastroenteritis |  | Per admission (hospitalisation > 1 day) | 152,179.40 | 6,996.00 |
| Bronchitis |  | Per admission (hospitalisation > 1 day) | 464,701.58 | 620,958.95 |
| Pulmonary tuberculosis |  | Per admission (hospitalisation > 1 day) | 499,598.52 | 264,771.33 |

Source: JMDC Claims Database .

1L, first line; 2L+, second or later line; ABA, abatacept; JPY, Japanese Yen; TNFi, tumour necrosis factor inhibitor.
