# Supplementary material for: Cost-consequence of abatacept as first-line therapy in Japanese rheumatoid arthritis patients using IORRA real-world data
Source: PLoS One. 2022 Nov 16;17(11):e0277566. doi: 10.1371/journal.pone.0277566 (PMC9668164; doi:10.1371/journal.pone.0277566)
Supplement: S9 Table — Source: JMDC Claims Database. 1L, first line; 2L+, second or later line; ABA, abatacept; JPY, Japanese Yen; TNFi, tumour necrosis factor inhibitor. (DOCX) [file pone.0277566.s010.docx]

**S9 Table. Disease monitoring costs.**

| Required resource |  | Unit | Cost (JPY)  ABA-1L vs. ABA-2L+ | Cost (JPY)  ABA-1L vs. TNFi-1L |
| --- | --- | --- | --- | --- |
| Radiographic exams |  | Per session | 2,658.82 | 2,608.71 |
| Outpatient visit |  | Per visit | 1,129.51 | 1,168.19 |
| Routine blood exams |  | Per visit | 597.15 | 583.37 |
| Inpatient visit |  | Per visit | 94,772.08 | 140,198.00 |

Source: JMDC Claims Database.

1L, first line; 2L+, second or later line; ABA, abatacept; JMDC, Japan Medical Data Center Inc; JPY, Japanese Yen; TNFi, tumour necrosis factor inhibitor.
