# Supplementary material for: Cost-consequence of abatacept as first-line therapy in Japanese rheumatoid arthritis patients using IORRA real-world data
Source: PLoS One. 2022 Nov 16;17(11):e0277566. doi: 10.1371/journal.pone.0277566 (PMC9668164; doi:10.1371/journal.pone.0277566)
Supplement: S10 Table — Ten most influential parameters on the difference in total costs (JPY). Minimum: -719,173, 388; Maximum: 1,040,202,718; Base case: -37,715,332. 1L, first line; ABA, abatacept; MTX, methotrexate; NSAID, non-steroidal anti-inflammatory drug; TNFi-, tumour necrosis factor inhibitor. (DOCX) [file pone.0277566.s011.docx]

**S10 Table. One-way sensitivity analysis: ABA-1L vs. TNFi-1L.**

**Ten most influential parameters on the difference in total costs (JPY)**

| **No** | **Parameter** | **Lower bound** | **Upper bound** | **Difference** |
| --- | --- | --- | --- | --- |
| 1 | Unit cost: TNFi-1L | 498,777,066 | −574,207,731 | 1,072,984,797 |
| 2 | Unit cost: ABA 1L | −563,726,296 | 488,295,631 | 1,052,021,928 |
| 3 | Incidence bronchitis: TNFi-1L | −18,889,036 | −59,392,764 | 40,503,727 |
| 4 | Incidence bronchitis: ABA 1L | −55,508,011 | −16,884,834 | 38,623,177 |
| 5 | Incidence SAE-related urinary tract infection: TNFi-1L | −27,027,816 | −52,663,157 | 25,635,342 |
| 6 | Incidence SAE-related urinary tract infection: ABA 1L | −46,797,137 | −24,201,210 | 22,595,926 |
| 7 | Sulfasalazine dose per day (mg): ABA 1L | −39,899,805 | −35,530,860 | 4,368,945 |
| 8 | Sulfasalazine duration (total days): ABA 1L | −39,899,805 | −35,530,860 | 4,368,945 |
| 9 | Prednisone duration (total days): TNFi-1L | −35,941,485 | −39,489,180 | 3,547,695 |
| 10 | Prednisone dose per day (mg): ABA 1L | −39,429,421 | −36,001,244 | 3,428,177 |

Minimum: −574,207,731; Maximum: 498,777,066; Base case: −37,715,332

1L, first line; ABA, abatacept; MTX, methotrexate; NSAID, non-steroidal anti-inflammatory drug; TNFi, tumour necrosis factor inhibitor.
