# Supplementary material for: Cost-consequence of abatacept as first-line therapy in Japanese rheumatoid arthritis patients using IORRA real-world data
Source: PLoS One. 2022 Nov 16;17(11):e0277566. doi: 10.1371/journal.pone.0277566 (PMC9668164; doi:10.1371/journal.pone.0277566)
Supplement: S11 Table — Ten most influential parameters on the difference in total costs (JPY). Minimum: -719,173, 388; Maximum: 1,040,202,718; Base case: 1,941,292. 1L, first line; 2L+, second or later line; ABA, abatacept; MTX, methotrexate; NSAID, non-steroidal anti-inflammatory drug. (DOCX) [file pone.0277566.s012.docx]

**S11 Table. One-way sensitivity analysis: ABA 1L vs. ABA 2L+.**

**Ten most influential parameters on the difference in total costs (JPY)**

| **No** | **Parameter** | **Lower bound** | **Upper bound** | **Difference** |
| --- | --- | --- | --- | --- |
| 1 | Unit cost: ABA 2L+ | 521,467,560 | −517,584,975 | 1,039,052,535 |
| 2 | Unit cost: ABA 1L | −514,455,733 | 518,338,317 | 1,032,794,049 |
| 3 | Incidence bronchitis: ABA 1L | −825,628 | 4,708,212 | 5,533,840 |
| 4 | Prednisone dose per day (mg): ABA 2L+ | 2,615,303 | −1,923,595 | 4,538,898 |
| 5 | Incidence bronchitis: ABA 2L+ | 4,203,885 | −321,300 | 4,525,185 |
| 6 | Sulfasalazine dose per day (mg): ABA 1L | −145,472 | 4,028,056 | 4,173,528 |
| 7 | Sulfasalazine duration (total days): ABA 1L | −145,472 | 4,028,056 | 4,173,528 |
| 8 | Unit cost: Pneumonia | 7,874,952 | 3,721,390 | 4,153,562 |
| 9 | Incidence SAE-related urinary tract infection: ABA 2L+ | 3,599,802 | 282,782 | 3,317,019 |
| 10 | Prednisone duration (total days): ABA 2L+ | 3,505,165 | 377,420 | 3,127,745 |

Minimum:−517,584,975; Maximum: 521,467,560; Base case: 1,941,292

1L, first line; 2L+, second or later line; ABA, abatacept; MTX, methotrexate; NSAID, non-steroidal anti-inflammatory drug.
